# Therapeutic approaches for chronic hepatitis C: a concise review

**DOI:** 10.3389/fphar.2023.1334160

**Published:** 2024-01-12

**Authors:** Allah Nawaz, Azhar Manzoor, Saeed Ahmed, Naveed Ahmed, Waseem Abbas, Mushtaq Ahmad Mir, Muhammad Bilal, Alisha Sheikh, Saleem Ahmad, Ishtiaq Jeelani, Takashi Nakagawa

**Affiliations:** ^1^ Joslin Diabetes Center, Harvard Medical School, Harvard University, Boston, MA, United States; ^2^ Department of Molecular and Medical Pharmacology, Faculty of Medicine, University of Toyama, Toyama, Japan; ^3^ Department of Surgery, Bahawal Victoria Hospital, Bahawalpur, Pakistan; ^4^ Department of Medicine, and Surgery, Rawalpindi Medical University, Rawalpindi, Punjab, Pakistan; ^5^ Department of Pharmacy, University of Poonch Rawalakot, Rawalakot, Azad Jammu and Kashmir (AJ&K), Pakistan; ^6^ Department of Clinical Laboratory Sciences, College of Applied Medical Sciences, King Khalid University, Abha, Saudi Arabia; ^7^ First Department of Internal Medicine, Faculty of Medicine, University of Toyama, Toyama, Japan; ^8^ Jammu Institute of Ayurveda and Research, University of Jammu, Jammu, India; ^9^ Cardiovascular Center of Excellence, Louisiana State University Health Sciences Center, New Orleans, LA, United States

**Keywords:** hepatitis C virus, direct-acting antiviral agents (DAAs), botanical drugs, hepatitis C, hepatoprotective and antiviral properties of medicinal plants

## Abstract

Hepatitis C virus (HCV) infection is a significant global health concern, prompting the need for effective treatment strategies. This in-depth review critically assesses the landscape of HCV treatment, drawing parallels between traditional interferon/ribavirin therapy historically pivotal in HCV management and herbal approaches rooted in traditional and complementary medicine. Advancements in therapeutic development and enhanced clinical outcomes axis on a comprehensive understanding of the diverse HCV genome, its natural variations, pathogenesis, and the impact of dietary, social, environmental, and economic factors. A thorough analysis was conducted through reputable sources such as Science Direct, PubMed, Scopus, Web of Science, books, and dissertations. This review primarily focuses on the intricate nature of HCV genomes and explores the potential of botanical drugs in both preventing and treating HCV infections.

## Background

Hepatitis C virus (HCV) infection is a significant global health issue and is regarded as one of the primary causes of mortality worldwide. According to the World Health Organization (WHO), the worldwide prevalence of HCV infections is currently reported to be 58 million, with an estimated 1.5 million new HCV infections occurring each year. This high incidence results in approximately 290,000 deaths annually attributed to HCV infection (WHO, 2022). While most cases of HCV infection remain asymptomatic, the disease can advance to chronic conditions like liver fibrosis, liver cirrhosis, hepatocellular carcinoma or liver failure. Notably, liver cirrhosis emerges in around 20% of individuals with chronic hepatitis C. Chronic liver disease is mainly attributed to excessive alcohol consumption and persistent infections with hepatitis B and C viruses (Singh and Hoyert, 2000). Several factors contribute to an elevated risk of HCV infection, including alcohol consumption, immunosuppression, and acquisition of HCV after the age of 40.

Recent research has elucidated that the impact of HCV extends beyond hepatocytes, leading to extrahepatic manifestations such as lymphoproliferative disorders, insulin resistance, type 2 diabetes, renal disease, and neurological disorders (Jacobson et al., 2010; Ito et al., 2011; Stanaway et al., 2016; Vanni et al., 2016; Drazilova et al., 2018; Moustafa et al., 2020). Consequently, there is an urgent need to identify new, and intensive treatment approaches capable of addressing both intra- and extrahepatic manifestations of HCV. The therapeutic approach for HCV is undergoing dynamic advancements aimed at attaining optimal responses and sustained viral eradication over the long term. The introduction of Interferon alpha-2b in 1986 marked an initial milestone in the pursuit of a curative approach for HCV (Tong et al., 1997; Hoofnagle et al., 1986). However, the sustained virologic response (SVR) rate with interferon monotherapy was limited to 10%–20% (Farrell et al., 1998; McHutchison et al., 1998). Subsequent research revealed that Ribavirin, an orally active synthetic guanosine analogue with antiviral and immunomodulatory properties, could enhance treatment outcomes when combined with interferon therapy (Bodenheimer et al., 1997; Davis et al., 1998). The administration of interferon and ribavirin therapy for the treatment of HCV can induce several adverse effects, including flu-like symptoms, nausea, vomiting, depression, insomnia, weight loss, anemia, and skin reactions. A significant advancement in HCV treatment has emerged with the introduction of various oral regimens that incorporate Direct-Acting Antivirals (DAAs), each characterized by distinct mechanisms of action (Asselah et al., 2018; Christensen et al., 2018). These treatments boast a favorable safety profile and are generally well-tolerated, resulting in a remarkable increase in SVR rates, often nearing 100%.

DAAs offer several advantages, including minimal side effects, short treatment durations (typically 8–12 weeks), and a low likelihood of viral resistance development. Targeting specific steps in the HCV life cycle, DAAs provide highly effective, well-tolerated, and curative treatment options. With cure rates often exceeding 95% across diverse patient populations and HCV genotypes, DAAs have a high barrier to the development of drug resistance. This approach has revolutionized HCV management, allowing healthcare providers to offer curative therapies to a broad range of patients, including those with co-infections (e.g., HIV) and special populations such as individuals with cirrhosis, transplant recipients, and people who inject drugs. While DAAs offer significant advantages, their high cost has raised concerns about access to treatment in some regions. Efforts are ongoing to make these medications more accessible globally.

In contrast, herbal treatments for HCV represent a traditional and alternative approach rooted in centuries-old natural remedies. Although some herbal products have been investigated for their hepatoprotective and potential antiviral properties, scientific evidence supporting their efficacy and safety in HCV management is limited and inconsistent. Herbs such as milk thistle, licorice root, and curcumin have been explored for potential benefits, but clinical study results are inconclusive. Moreover, the lack of standardization and quality control in herbal products raises concerns about consistency and safety.

### Epidemiology of HCV worldwide

On a global scale, chronic liver disease and cirrhosis rank as the 10th leading cause of death. This condition affects both males and females disproportionately between the ages of 35 and 64, making it the 5th leading cause of mortality among individuals aged 45 to 64. Approximately 70% of individuals chronically infected with HCV develop progressive liver disease, and HCV infection accounts for the chronic liver disease in 40% of all affected patients (Nainan et al., 2006). According to the World Health Organization (WHO), approximately 200 million people are currently living with HCV, with an annual incidence of 3.5–4.0 million new infections. The prevalence of HCV varies globally, with notable regional distinctions. These insights into the global epidemiology of HCV underscore the importance of comprehensive strategies for prevention, diagnosis, and management on a worldwide scale.

## HCV-induced tissue damage via activation of complement system

**FIGURE 1:**
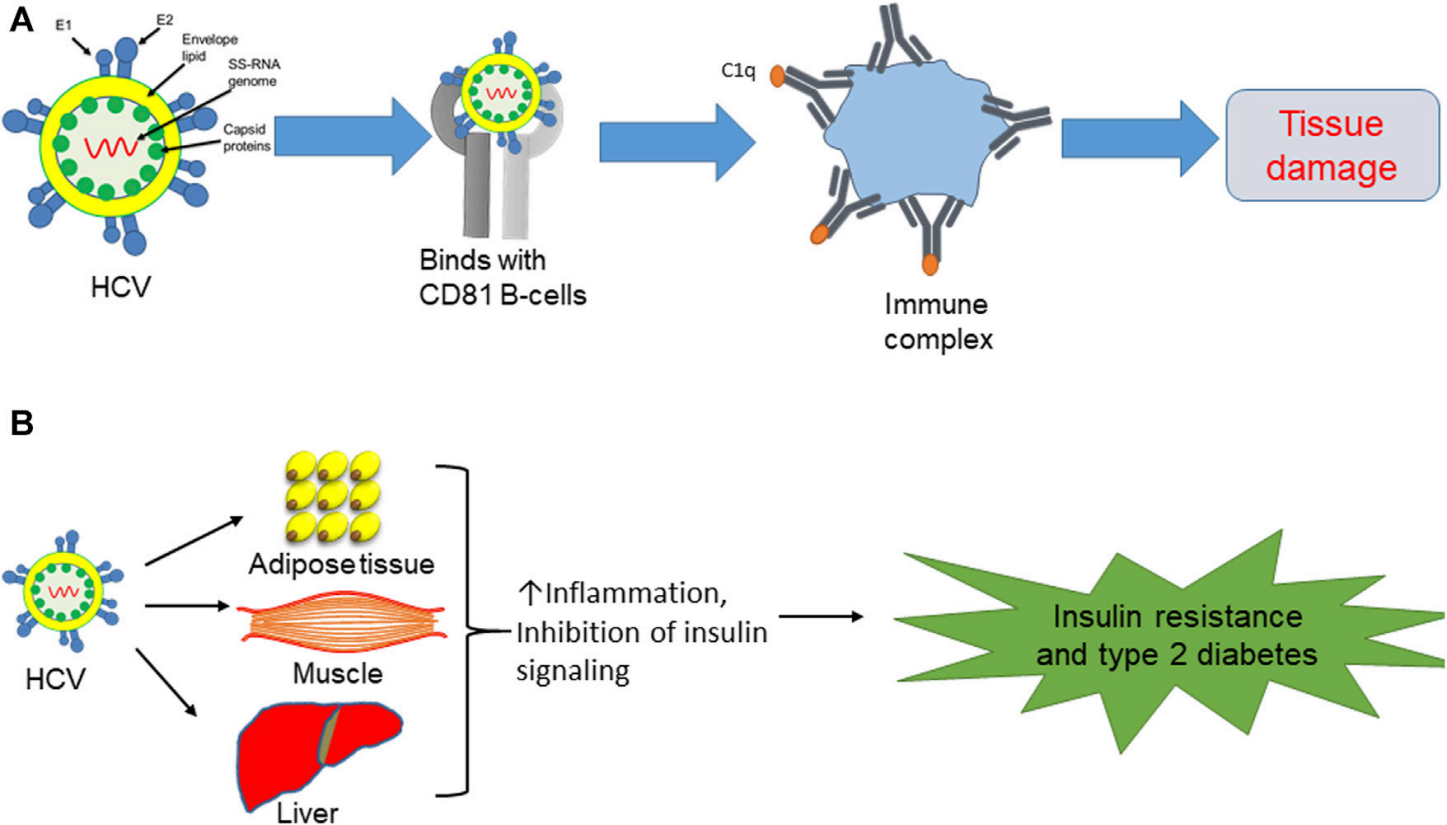
**(A)** HCV binds with CD81 B cells, leading to antibody production and formation of immune complexes, which then initiate tissue damage. **(B)** Direct effect of HCV on insulin resistance and type 2 diabetes via induction of proinflammatory cytokines in adipose tissue and liver, while also blocking insulin signaling in muscle.

The liver, the largest solid organ in the body, plays a pivotal role in diverse physiological functions. Comprising various cell types, the liver includes hepatocytes as the predominant cell type, along with non-parenchymal cells, hepatic stellate cells, endothelial cells, and Kupffer cells. The latter are resident macrophages that mainly regulate liver homeostasis during liver inflammation (Kazankov et al., 2019; Sakai et al., 2019; Alharthi et al., 2020). Immune cells are key players in various metabolic diseases, including insulin resistance, fatty liver, and atherosclerosis. HCV, upon binding to extrahepatic peripheral B cells (CD81), triggers dysregulation within the immune system. This infection results in the chronic stimulation of lymphocytes, ultimately leading to the expansion of B-cell clones. This process then activates the complement system and antibody production to form immune complexes, resulting in tissue damage (Jacobson et al., 2010; Ito et al., 2011; Moustafa et al., 2020) (Figure 1A).

## HCV and extrahepatic manifestations of metabolic disorders

HCV is involved in extrahepatic manifestations of metabolic disorders, particularly insulin resistance and type 2 diabetes, due to the aberrant activation of inflammatory cytokines. In the context of chronic HCV infections, there is a notable production of inflammatory cytokines, including interleukin-6 (IL-6) and tumor necrosis factor-alpha (TNF-α), within the liver. These cytokines, in turn, disrupt insulin signaling pathways, leading to the development of insulin resistance. Beyond the heightened risk of type 2 diabetes associated with HCV infections, individuals with chronic HCV infections also exhibit an elevated prevalence of cardiac-cerebrovascular disorders. This intricate relationship underscores the systemic impact of HCV beyond the liver and emphasizes the potential role of viral-induced inflammatory responses in contributing to broader metabolic and cardiovascular complications (Drazilova et al., 2018; Moustafa et al., 2020). HCV infection seems to accelerate the inflammatory response and hinder insulin signaling in metabolically responsive tissues, including the liver, adipose tissue, and skeletal muscle (Figure 1B). The dysregulation of inflammatory pathways and interference with insulin signaling in these tissues contribute to the development of metabolic disturbances, including insulin resistance, highlighting the systemic effects of HCV infection beyond the liver (Fujisaka et al., 2009; Fujisaka et al., 2013; Fujisaka et al., 2016; Nawaz et al., 2017a; Nawaz et al., 2017b; Takikawa et al., 2019). Indeed, aging-induced inflammation plays a substantial role in the onset of diverse disorders, including diabetes, atherosclerosis, and mitochondrial dysfunction. The inflammatory milieu associated with aging can amplify the overall impact of HCV infection on the body’s health, exacerbating its effects on metabolic and physiological processes (Zhang et al., 2016; Chia et al., 2018; Kazankov et al., 2019; Luo et al., 2019; Reidy et al., 2019). Consequently, HCV infection is correlated with metabolic complications due to the abnormal activation of inflammatory cytokines.

## Contribution of botanical drugs in HCV management

The use of various medicinal plants for managing chronic hepatitis C infections has been a part of traditional medicine in different cultures for centuries. Many societies have relied on natural remedies derived from plants to address various health issues, including liver ailments. It is important to note that while traditional practices often involve the use of medicinal plants, the effectiveness and safety of these remedies can vary, and scientific research is essential to validate their therapeutic potential. Botanical drug(s), individually or in combination, are effective against HCV infection, blocking the virus’s entry, translation, replication, and assembly (summarized in Table 1). Molecular studies have also demonstrated that medicinal plants can be used to develop anti-HCV drugs. Silymarin, derived from the seeds of the milk thistle plant (Silybum marianum: family Asteraceae), is known to inhibit inflammatory cytokines and other transcriptional factors, viral entry into hepatocytes, and viral replication (Polyak et al., 2007). Quercetin is reported to block HCV replication via a direct inhibitory effect on NS3 polymerase and IRES activity (Gonzalez et al., 2009; Bachmetov et al., 2012). *Ladanein* and *Limonium sinense* block viral entry through effects on post-attachment entry steps, including uncoating, fusion, and endocytosis (Haid et al., 2012; Hsu et al., 2015; Jardim et al., 2018). *Magnolia officinalis,* a member of *Magnoliaceae* family, blocks HCV translation (Lam et al., 2016). Furthermore, *glycyrrhizin,* an inherent compound present in the roots of the *Glycyrrhiza glabra* plant from the *Fabaceae* family, demonstrates anti-HCV activity. It acts specifically by impeding viral translation and replication, leading to a reduction in viral titer (Ashfaq et al., 2011; Matsumoto et al., 2013; Ashfaq and Idrees, 2014; Li et al., 2014; Li et al., 2019).

**TABLE 1:** Compilation of medicinal plants and their metabolites, along with potential mechanisms of action against HCV.

Medicinal plants/Metabolites	Effect on HCV	Properties	Mechanism	References
Milk Thistle (Silybum marianum): Asteraceae family	Viral entry, Viral replication	Hepatoprotective, anti-inflammatory	Inhibition of core protein and NS5 RNA-dependent RNA polymerase	Polyak et al. (2007), Polyak et al. (2010), Wagoner et al. (2010), Ahmed-Belkacem et al. (2010), Lovelace et al. (2015)
Diosgenin: A naturally occurring steroid sapogenin present in certain plants including Trigonella foenum-graecum (Family: Fabaceae), Costus speciosus (Family: Costaceae), Tribulus terrestris L (Family: Zygophyllaceae), Smilax china L. (Family: Smilacaceae), Rhizoma polgonation (Family: Asparagaceae)	Viral replication	Antiviral	Inhibition of transcription factor 3 and signal transducer	Wang et al. (2011), Jesus et al. (2016)
Embelia ribes: Primulaceae family	Viral replication	Antiviral	Inhibition of IRES activity and NS3 polymerase	Gonzalez et al. (2009), Bachmetov et al. (2012)
Iridoids: Secondary metabolites in species belonging to the Apocynaceae, Lamiaceae, Loganiaceae, Rubiaceae, Scrophulariaceae and Verbenaceae families	Viral entry	Antiviral	Blockage of E2 and CD81 contact	McGarvey et al. (1988), Zhang et al. (2009)
Luteolin, from the plant Reseda luteola: Resedaceae family	Viral replication	Antiviral	Inhibition of NS5B polymerase activity	Liu et al. (2012), Shibata et al. (2014), Zakaryan et al. (2017)
Naringenin: widely distributed in several Citrus fruits, bergamot, tomatoes and other fruits	Viral assembly	Antiviral	Suppression of core protein activity	Nahmias et al. (2008), Cheung et al. (1988)
Camellia sinensis: Theaceae	HCV replication and viral assembly	Antioxidant, anti-inflammatory, immunomodulatory	Direct antiviral effects against HCV are not well-established. May interfere with activity of NS3 and 4A proteases	Calland et al. (2012b), Calland et al. (2012a), Chen et al. (2012), Haid et al. (2012), Wang et al. (2016), Mekky et al. (2019)
Mangnolia grandiflora: Magnoliaceae family Swietenia macrophylla: Commonly known as mahogany, is a species of tropical hardwood tree belonging to the Meliaceae family. Phyllanthus amarus: Phyllanthus amarus, also known as “bhui amla” or “stonebreaker,” is a tropical plant belonging to the Phyllanthaceae family. *Excoecaria agallocha*: Commonly known as the “Blinding Tree” or “Milky Mangrove,” is a species of flowering plant in the Euphorbiaceae family	May block viral replication and viral assembly	Hepatoprotective, antiviral efficacy against HCV	-	Lan et al. (2012), Wu et al. (2012), Ravikumar et al. (2011), Bokesch et al. (2003), Takebe et al. (2013), Choi et al. (2014), Li et al. (2012)
Ladanein: is a metabolite found in certain plants, particularly in species belonging to the Labiatae (mint) family	Viral entry, Post attachment entry step	Hepatoprotective	Inhibition of receptor interactions, virus endocytosis, or membrane fusion	Haid et al. (2012), Hsu et al. (2015), Jardim et al. (2018)
Magnolia officinalis: Magnoliaceae family	HCV translation	Hepatoprotective, anti-inflammatory	-	Lam et al. (2016)
Glycyrrhizin: is a natural compound found in the root of the licorice plant (Glycyrrhiza glabra), which belongs to the legume family (Fabaceae)	Viral translation and replication	Antiviral, anti-inflammatory	-	Ashfaq et al. (2011), Ashfaq and Idrees, (2014), Li et al. (2014), Li et al. (2019), Matsumoto et al. (2013)

We previously found that a polyherbal formulation comprising five medicinal plants, including *Silymarin,* showed comparable results to interferon and ribavirin therapy in reducing viral loads in patients with HCV (Nawaz et al., 2015; Nawaz et al., 2016). In addition, polyherbal formulation has been found to have minimal or no side effects and was well-tolerated by patients, while also contributing to an improvement in quality of life. A recent cross-sectional study conducted on patients with HCV infection showed that herbal medicines are safe and effective against HCV (Nsibirwa et al., 2020). Complementary and alternative medicine therapies (CAM) may offer potential benefits in alleviating the chronic liver disease associated with HCV, even though they may not directly inhibit or eliminate the viral infection itself. Some CAM therapies have shown biological effects, including antioxidant, anti-fibrotic, or immune-modulating activities, which could contribute to the amelioration of the disease (Ferenci et al., 1989). Here are some host factors targeted by CAM therapies that have been studied in the context of viral infections: 1) Immune system modulation: CAM therapies such as herbal supplements, vitamins, and minerals are often used with the aim of modulating the immune system. For example, certain herbs like echinacea (Asteraceae family) and astragalus (Fabaceae family) are believed to have immune-enhancing properties. 2) Some CAM approaches involve the use of herbs and supplements with purported antiviral effects. Examples include elderberry, garlic, and licorice root, which have been studied for their potential to inhibit viral replication. 3) Stress can negatively impact the immune system, making the body more susceptible to infections. Mind-body practices such as meditation, yoga, and acupuncture, which fall under the CAM umbrella, aim to reduce stress and promote overall wellbeing. 4) CAM often emphasizes the importance of a balanced and nutrient-rich diet to support overall health. Nutritional interventions, including dietary supplements and specific diets, may be recommended to enhance the host’s nutritional status, potentially supporting the immune response. 5) Probiotics, considered as CAM intervention, focus on supporting a healthy balance of gut bacteria. Since the gut microbiome plays a role in immune function, some CAM practitioners recommend probiotics to enhance the body’s defense mechanisms. It is important to note that the scientific evidence supporting these interventions can vary, and further research is often needed for validation.

**FIGURE 2:**
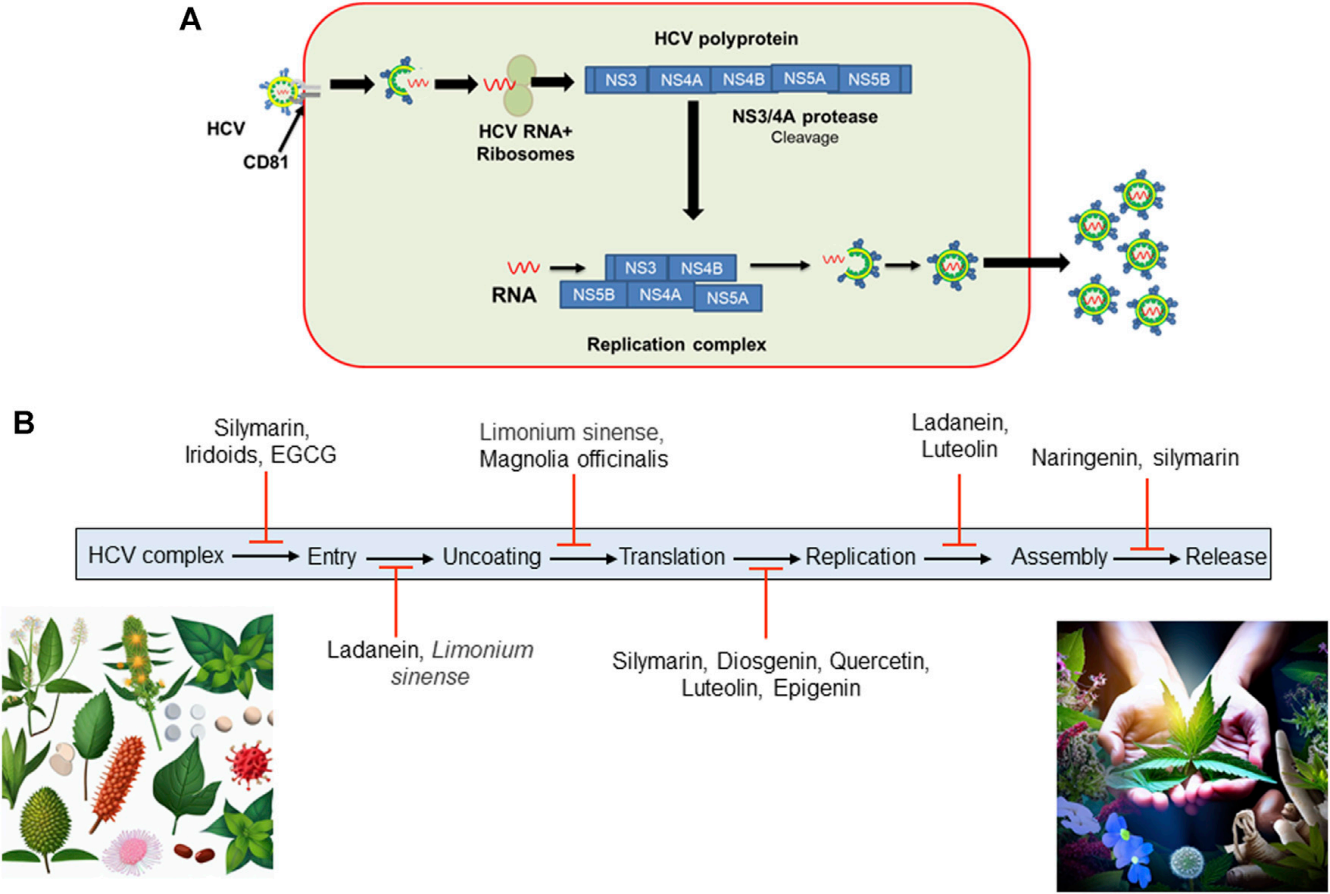
**(A)** HCV entry into hepatocytes. HCV complex enters into hepatocytes and uses a CD81 B-cell as a reservoir. After entry and fusion, the viral genome is released into the cytosol, accompanied by translation and replication. Once replication is complete, HCV assembles a new viral coat and is released from the host cell to infect other cells. **(B)** Certain botanical drugs have been documented to impede HCV activity by disrupting its replication cycle at various stages. (EGCG, Epigallocatechin-3-gallate found in green tea extract; HCV, Hepatitis C Virus).

In China, a combination of seven botanical drugs known as Sho-Sai-Ko or xiao-chai-hu-lang has been used to treat hepatitis C, leading to improvements in liver pathogenicity among selected hepatitis C patients who were not suitable candidates for interferon-based treatments (Deng et al., 2011). Hence, alternative herbal therapy presents a promising option for the treatment of hepatitis C (Nawaz et al., 2015). In Egypt, a year-long randomized double-blind trial was conducted with Silymarin on patients diagnosed with HCV. Despite the safe administration of Silymarin throughout the trial period, the study yielded discouraging outcomes with no significant improvements observed in terms of HCV viral load and serum ALT levels (Tanamly et al., 2004). On the contrary, Hepcinal, a formulation comprising Silymarin alongside four other drugs, exhibited enhanced clinical, biochemical, and serological responses (Nawaz et al., 2016). Shifting our focus to herbal treatments, we delve into their historical roots and significance in HCV management. Prominent herbs such as licorice root (*Glycyrrhiza glabra, Fabaceae family*), and curcumin (*Curcuma longa L. (turmeric)* of ginger family (*Zingiberaceae*)) have been subject to scientific scrutiny for their potential hepatoprotective and antiviral properties. Metabolites including flavonoids, alkaloids, and polyphenols present in *Phyllanthus niruri L. (Phyllanthaceae family)*, *Glycyrrhiza glabra* (licorice), and *Silybum marianum L.) Gaertn* (milk thistle) (Asteraceae family) have shown potential in inhibiting viral replication. Many medicinal plants including *Schisandra chinensis* (Schisandraceae family) and *Picrorhiza kurroa* (Plantaginaceae family), possess hepatoprotective properties, protecting liver from further damage. Some herbs, such as *Curcuma longa* (turmeric) (Zingiberaceae family), exhibit anti-inflammatory property that may help to mitigate the immune response and reduce liver inflammation. Overall, botanical drug(s), individually or in combination, are effective against HCV infection, blocking viral entry, translation, replication, and assembly. Furthermore, we acknowledge the challenges posed by the lack of standardization and quality control in herbal products. However, it is crucial to emphasize the need for further pharmacological investigations and large-scale clinical trials to validate the clinical safety and efficacy of these botanical drugs. We have summarized the effectiveness of selected botanical drugs and their ability to inhibit HCV activity in Figures 2A,B. In summary, while some botanical drugs may offer potential benefits for liver health, their use for HCV treatment should be approached with caution. Consultation with a healthcare provider and adherence to conventional medical treatments are essential for managing HCV effectively. As far as our current knowledge extends, there are no large-scale cross-sectional studies available that comprehensively demonstrate the clinical and serological outcomes of botanical drugs in treating HCV. However, in smaller-scale studies, researchers have documented the potential benefits of medicinal plants in combating HCV infection and have elucidated some of the molecular mechanisms involved. Additional research is needed to further understand the mechanisms underlying these improvements resulting from herbal treatments, as well as to provide additional evidence regarding their effectiveness and safety.

## Future perspectives and limitations

The emergence of new therapeutic approaches holds promise for curing a greater number of HCV patients. The availability of potent natural or botanical drugs for HCV infection is a positive development. Consequently, there should be a heightened focus on the screening and identification of potent medicinal plants for the management and treatment of both acute and chronic HCV infections. This exploration of botanical drugs may lead to more effective and accessible treatments for individuals affected by HCV.
